# Serine protease CrKP43 interacts with MAPK and regulates fungal development and mycoparasitism in *Clonostachys chloroleuca*

**DOI:** 10.1128/spectrum.02448-23

**Published:** 2023-10-13

**Authors:** Binna Lv, Xue Zhao, Yan Guo, Shidong Li, Manhong Sun

**Affiliations:** 1 Institute of Plant Protection, Chinese Academy of Agricultural Sciences, Beijing, China; Stony Brook University, Stony Brook, New York, USA

**Keywords:** *Clonostachys chloroleuca*, serine protease, mycoparasitism, MAPK, biocontrol

## Abstract

Serine proteases are a group of important hydrolytic enzymes that play vital roles in various cellular processes in fungi. In this study, the S8 serine protease-encoding gene *CrKP43* was identified in the highly efficient *Clonostachys chloroleuca* 67–1 (formerly *C. rosea* 67–1) strain, which was markedly upregulated when parasitizing *Sclerotinia sclerotiorum*. The function of *CrKP43* was investigated using gene deletion and complementation, and the results indicated that the lack of *CrKP43* resulted in deformed fungal hyphae and cell morphology, inhibition of conidiation, and decreased antagonistic activity toward the pathogenic fungus *Fusarium oxysporum* f. sp. *cucumerinum*. Moreover, the mutants displayed much weaker mycoparasitic ability to *S. sclerotiorum* sclerotia and lower control efficiency against soybean *Sclerotinia* rot compared with the wild-type strain. All biological characteristics and biocontrol activities were recovered when the *CrKP43* gene was reinserted into the fungus. Using qRT-PCR analysis and protein-protein interaction assays, it was further proved that the CrKP43 protein interacted with the mitogen-activated protein kinase (MAPK) Crmapk, suggesting serine proteases might be involved in the mycoparasitism of *C. chloroleuca* with the regulation of Crmapk. The findings improve our knowledge of serine proteases and their regulation in mycoparasites and help to illuminate the mechanisms underlying mycoparasitism of *C. chloroleuca*.

**IMPORTANCE:**

Mycoparasites play important roles in the biocontrol of plant fungal diseases, during which they secret multiple hydrolases such as serine proteases to degrade their fungal hosts. In this study, we demonstrated that the serine protease CrKP43 was involved in *C. chloroleuca* development and mycoparasitism with the regulation of Crmapk. To the best of our knowledge, it is the first report on the functions and regulatory mechanisms of serine proteases in *C. chloroleuca*. Our findings will provide new insight into the regulatory mechanisms of serine proteases in mycoparasites and contribute to clarifying the mechanisms underlying mycoparasitism of *C. chloroleuca*, which will facilitate the development of highly efficient fungal biocontrol agents as well.

## INTRODUCTION

Proteases (peptidases) are a group of hydrolytic enzymes that cleave peptide bonds of proteins to generate small peptides or amino acids (1, 2). Proteases are classified into families and clans based on the similarity of amino acid sequences and catalytic mechanisms, among which more than a third are serine proteases possessing an active site serine nucleophile that attacks carbonyl groups of substrates (3 – 5). According to the peptidase database MEROPS (http://merops.sanger.ac.uk), serine proteases of the clan subtilases (SB) are divided into two families: S8 (subtilisin-like proteinases) and S53 (serine-carboxyl proteinases) (6, 7). The S8 family includes the serine endopeptidase subtilisin and related enzymes with a conserved Asp-His-Ser catalytic triad (5, 8) that is very similar to the His-Asp-Ser catalytic triad present in S1, the most common protease family (9 – 11). These serine peptidases are widely used in daily chemical, food, and pharmaceutical industries and also in waste management (1, 12), but their application in the biological control of plant diseases remains limited (13, 14).

Serine proteases are broad-spectrum degradation enzymes that have been found in almost all organisms. In filamentous fungi, serine proteases are associated with infection, penetration, and colonization and are considered key virulence factors in fungal pathogenicity (6, 15). The best-known serine protease in biocontrol fungi is the cuticle-degrading protease Pr1 derived from the entomopathogenic fungus *Beauveria bassiana* (5, 16). Entomopathogenic fungi are capable of degrading insect cuticles by secreting various enzymes, including serine proteases and other hydrolases such as chitinases, proteases, and lipases, as virulence determinants (15). Extracellular serine proteases secreted by nematophagous fungi, such as *Paecilomyces lilacinus* and *Pochonia chlamydosporia*, are capable of degrading the cuticles of plant nematodes, which helps the parasites to penetrate their hosts (17 – 19). In mycoparasites, serine proteases are also frequently involved in colonization of other fungi (20).

When hosts are infected, the integrity of the cell wall is compromised, which facilitates penetration of the antagonists. The genes *P8048* and *P10261* encoding two S8 family peptidases in *Trichoderma harzianum* were suggested to be involved in mycoparasitism by digesting structural proteins of the host (14). The gene *tvsp1* orthologous to *P10261* is also essential for mycoparasitism and biocontrol activities in *Trichoderma virens*; overexpression of *tvsp1* improved the ability of the fungus to protect cotton seedlings against infection by *Rhizoctonia solani* (21). Moreover, the serine protease gene *sprt* belonging to the S8 family was found to be upregulated in *T. harzianum* when encountered with *S. sclerotiorum*, which assisted fungal colonization by penetrating the surface of sclerotia and apothecia (22). The S8 serine protease gene *prs6* in *C. rosea* strain IK726 was induced during interaction with the phytopathogen *Fusarium graminearum* (23). However, the regulatory mechanisms of serine proteases in biocontrol fungi have not been fully elucidated.

Parasitism by biocontrol fungi is a complex process involving recognition, degradation of cell walls, and subsequent infection (24). Several serine proteases in *Trichoderma* species are associated with their responses to pathogens (13, 24, 25). In *T. harzianum*, the serine protease-encoding gene *ser* is vital in the early stages of interaction with *Fusarium solani* (13), and the protease gene *prb1* is induced before hyphae come into contact with *R. solani* (26). Once mycoparasites perceive external stimuli, a series of signal transduction pathways are initiated, and sequential cellular responses are triggered (27). Mitogen-activated protein kinase (MAPK) pathways are important molecular systems that directly connect signal transduction and cell responses to various stresses via phosphorylation cascade reactions (28). In our previous studies, the MAPK-encoding gene *Crmapk* was found to be of great importance in mycoparasitism of the efficient *Clonostachys chloroleuca* isolate 67–1 (29), and 60 proteins potentially interacting with Crmapk were identified from a yeast two-hybrid (Y2H) library of the 67–1 strain (30), among which the S8 serine protease CrKP43 was strongly linked to mycoparasitism regulated by Crmapk in *C. chloroleuca*.

*C. chloroleuca* (formerly classified as *C. rosea*), a member of the order *Hypocreales* in the class *Sordariomycetes*, is an important mycoparasite of a range of pathogenic fungi including *S. sclerotiorum*, *R. solani,* and *Botrytis cinerea* (31 – 33), and it has shown excellent biocontrol effects in controlling various plant fungal diseases in greenhouse and field (34, 35). The biocontrol mechanisms of *C. chloroleuca* are primarily attributed to mycoparasitism, secretion of cell wall-degrading enzymes, production of secondary metabolites such as antibiotics and indole alkaloids, and induction of plant resistance (35 – 39). Several genes associated with transcription factors, secreted proteins, secondary metabolites, signal transduction, and membrane transporters are involved in the mycoparasitism and biocontrol activities of *C. chloroleuca* (34, 37, 40, 41). However, the roles of S8 proteases and their regulation in *C. chloroleuca* during the mycoparasitic process have not yet been investigated.

In the present study, the S8 serine protease CrKP43 in *C. chloroleuca* 67–1 strain was identified, and its roles in fungal growth, conidiation, and biocontrol activities were explored. Protein-protein interaction assays showed that CrKP43 interacted with the MAP kinase Crmapk, suggesting that CrKP43 might be associated with the MAPK pathway, which is involved in the mycoparasitism of *C. chloroleuca* (29). This study provides valuable clues to reveal the regulation of serine proteases in mycoparasitism and improve our understanding of the mechanisms underlying the biocontrol of *C. chloroleuca*.

## RESULTS

### Identiﬁcation of CrKP43 from *C. chloroleuca*

**Fig 1:**
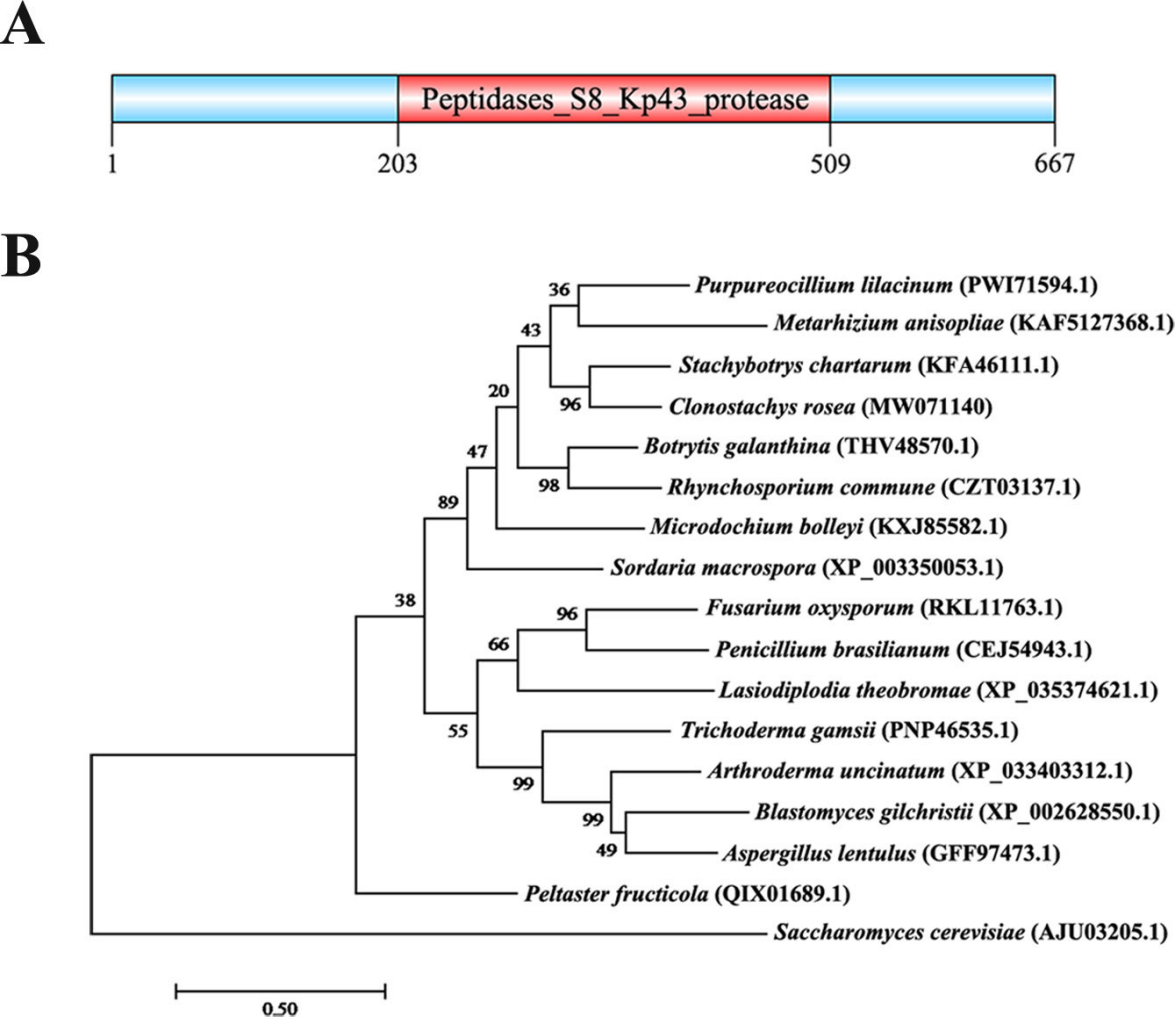
Characterization of the CrKP43 protein in *C. chloroleuca* 67–1. (**A**) Domain structure of CrKP43 annotated by SMART MODE (http://smart.embl.de/). (**B**) Phylogenetic analysis of CrKP43 and its homologs in other fungi. Amino acid sequences were aligned using Clustal X and analyzed using MEGA 7.0 with the maximum likelihood method. Identifications in parentheses indicate the GenBank accession numbers, and numbers at nodes represent the bootstrap values of 1,000 replicates. The bars represent the sequence divergence (bar = 0.50).

**Fig 2:**
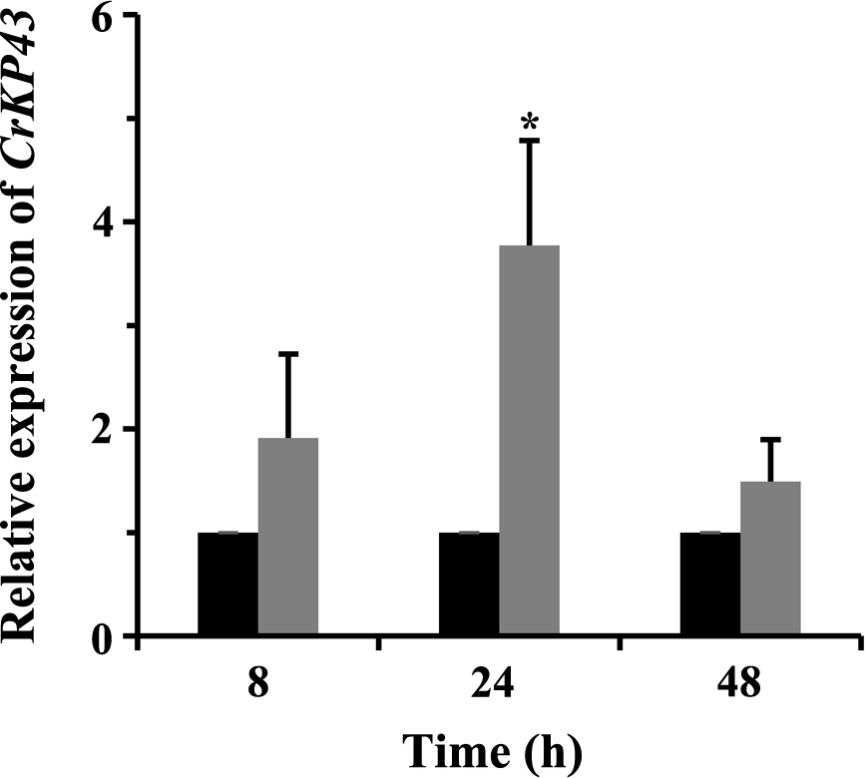
Expression levels of *CrKP43* in *C. chloroleuca* under the induction of *S. sclerotiorum* sclerotia. The gray columns represent the *C. chloroleuca* samples treated with fresh sclerotia, while the black columns represent the controls without sclerotia. Relative expression levels of *CrKP43* were calculated using the 2^–∆∆Ct^ method, and statistical analyses were carried out by Tukey’s multiple comparison tests. The error bars show the standard deviations of three replicates, and asterisks represent the signiﬁcant differences (*P* < 0.05).

The gene sequence of *CrKP43* (GenBank accession number: MW071140) was cloned from the draft genome sequence of *C. chloroleuca* 67–1. The full-length *CrKP43* gene is 2,060 bp with one intron, encoding a protein of 667 amino acids. The amino acid sequence of the protein was predicted using the SMART prediction server, and a Kp43 domain, which belongs to the S8 family of serine peptidases, was detected (Fig. 1A). Phylogenetic analysis and sequence alignment of *CrKP43* with other fungal species revealed a close relatedness to its homolog in *Stachybotrys chartarum* and high conservation among various fungi (Fig. 1B). Quantitative reverse-transcription PCR (qRT-PCR) analysis of gene expression during the mycoparasitic process indicated that *CrKP43* was upregulated in *C. chloroleuca* throughout mycoparasitism, particularly at 24 h, and expression levels were more than 3.8-fold higher than those in the control (Fig. 2), consistent with transcriptome data from *C. chloroleuca* parasitizing *Sclerotinia sclorotiorum* (25).

### Deletion and complementation of *CrKP43*

To elucidate the biological functions of *CrKP43* in *C. chloroleuca*, gene deletion mutants were generated using a homologous recombination strategy (Fig. S1A). Among the 136 hygromycin-resistant transformants, four Δ*CrKP43* strains with identical phenotypic characteristics were verified by PCR analysis using primers CrKP43-IN-F/R (within the target gene), CrKP43-YZ-F/R (outside the homologous fragment), HPH-F/R (both ends of the *hph* gene), and CrKP43-YZ-F/HPH-R (Fig. S1B). The fragments amplified by the primer pair CrKP43-YZ-F/R were sequenced, indicating that the *CrKP43* gene was successfully replaced with a hygromycin B resistance cassette, as expected. For complementation of *CrKP43*, the vector pKN-CrKP43-C was transformed into the mutant strain, and seven corresponding Δ*CrKP43-C* complementation strains were obtained. RT-PCR verification demonstrated a complete loss of the *CrKP43* transcript in the Δ*CrKP43* mutants, while specific products were detected in the wild-type and complementation strains (Fig. S1C). These results suggest that the knockout of *CrKP43* was efficient, and the mutants could be used for further morphological analysis.

### CrKP43 affects *C. chloroleuca* hyphal morphology and conidiation

**Fig 3:**
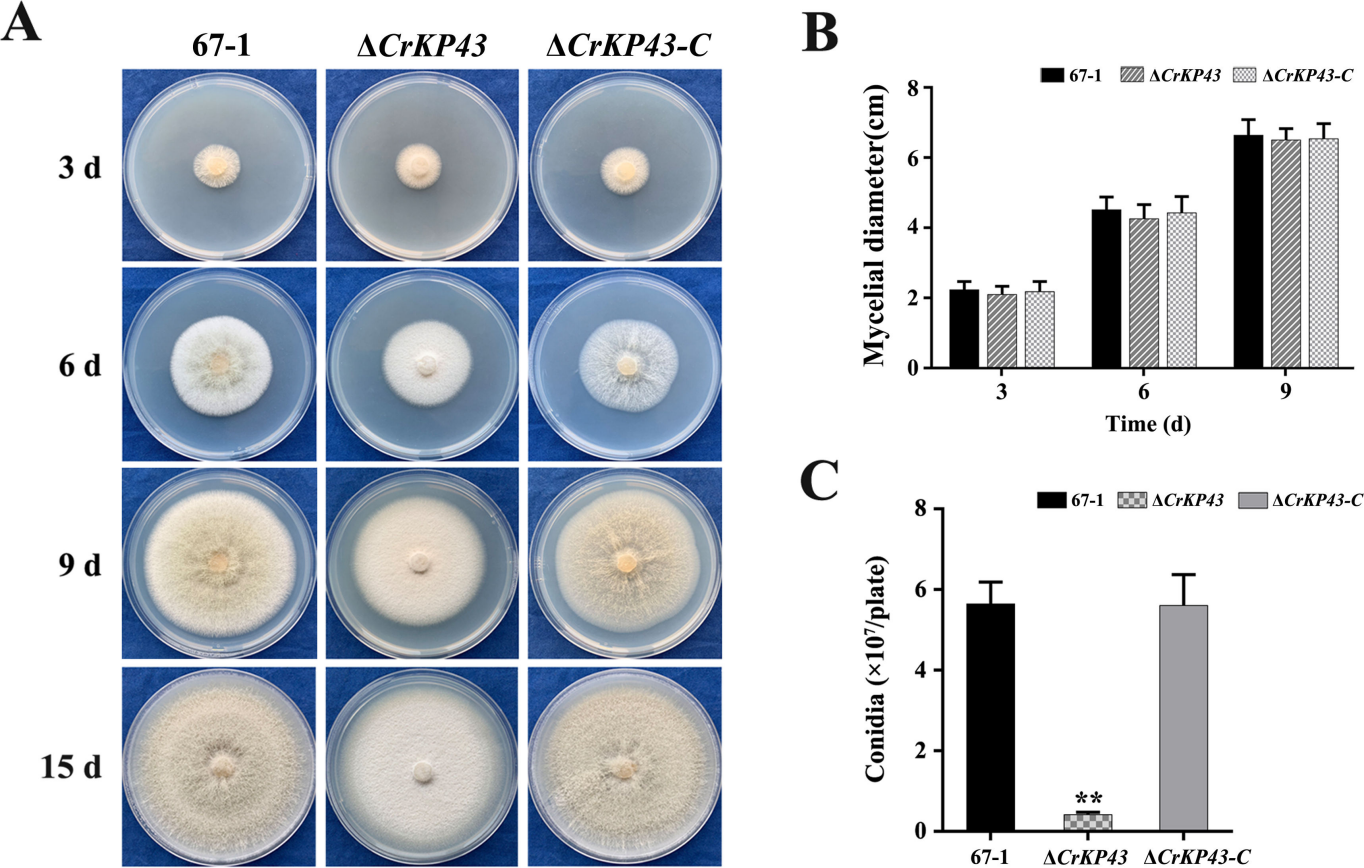
Effects of *CrKP43* on growth and conidiation of *C. chloroleuca* 67–1, Δ*CrKP43,* and Δ*CrKP43-C*. (**A**) Mycelial growth on PDA medium after 3, 6, and 9 days of incubation and conidiation after 15 days. (**B**) Statistical analysis of colony diameters in (**A**). (**C**) Conidia production on PDA plates. The results are the means of the three Δ*CrKP43* and Δ*CrKP43-C* mutants, and the means and standard errors were calculated from the three independent replicates. Statistical analyses were carried out using Tukey’s tests for multiple comparisons, and asterisks indicate the significant differences (*P* < 0.05).

Three Δ*CrKP43* and Δ*CrKP43-C* mutants were selected to explore the functions of the *CrKP43* gene. Observation of colony morphology showed that the mycelia of Δ*CrKP43* mutants were a little less compared to those of the wild-type 67–1 strain and complemented transformants; however, their mycelial extension rates were not significantly different (Fig. 3A and B). When the *CrKP43* gene was knocked out, conidiation decreased significantly. After being grown on potato dextrose agar (PDA) for 15 days, only 4.2 × 10^6^ spores/plate were harvested in Δ*CrKP43*, compared with 5.7 × 10^7^ and 5.6 × 10^7^ spores/plate in the wild-type and complementary strains (*P* < 0.01, Fig. 3C).

### CrKP43 has no impact on *C. chloroleuca* stress tolerance

The sensitivities of *C. chloroleuca* isolates to osmolarity, oxidative stress, and cell membrane/wall stresses were consistent. When NaCl (1 M), KCl (1 M), glycerin (1 M), sorbitol (1 M), H_2_O_2_ (20 mM), SDS (0.03%), and Congo Red (0.3 mg/mL) were added, the growth of the wild-type 67–1, Δ*CrKP43,* and Δ*CrKP43-C* strains was similar, indicating that the *CrKP43* gene is not involved in the *C. chloroleuca* responses to environmental stresses (Fig. S2).

### CrKP43 is required for normal cellular morphology of *C. chloroleuca*

**Fig 4:**
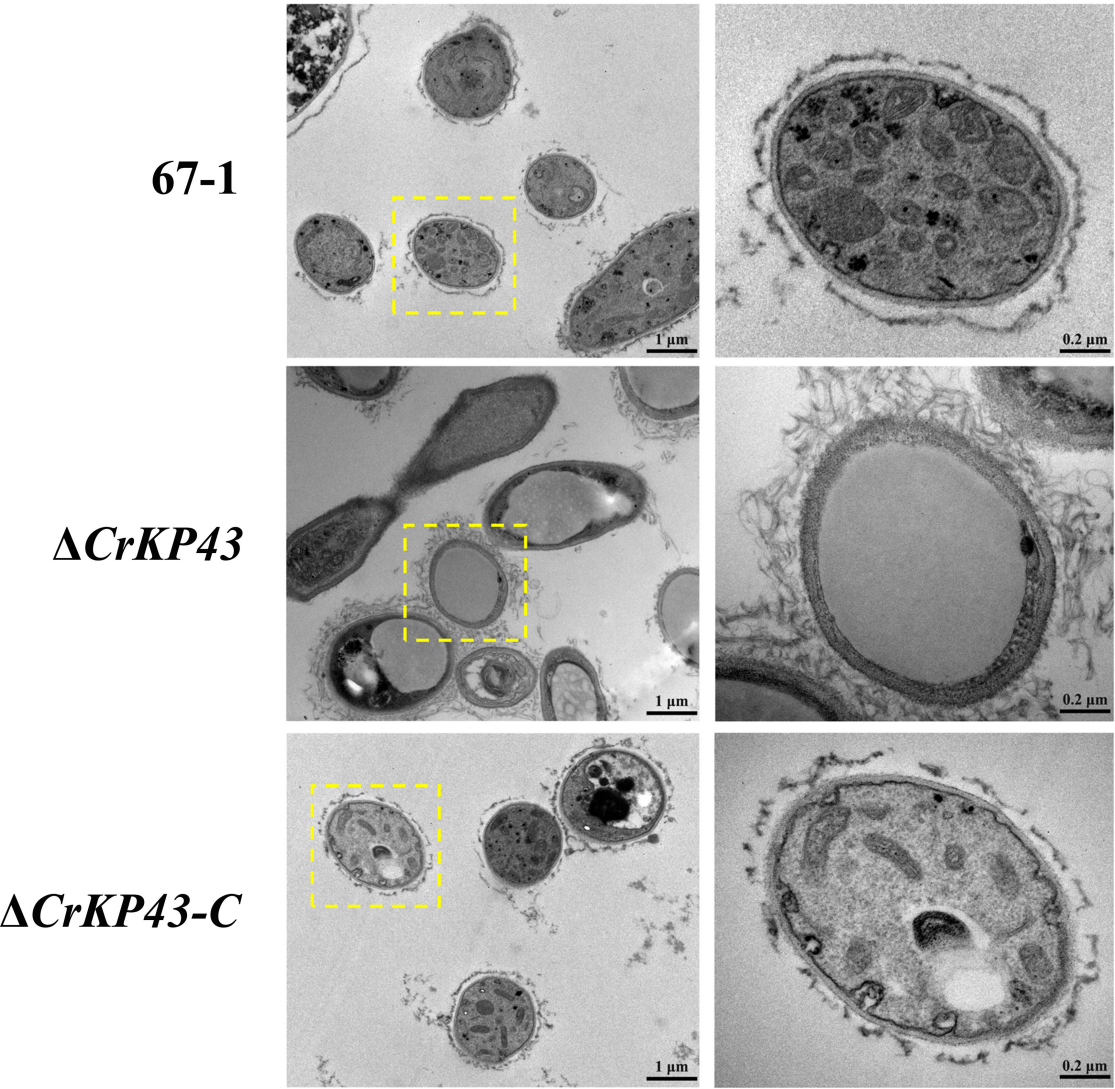
Impacts of *CrKP43* on the cellular morphology of *C. chloroleuca* 67–1, Δ*CrKP43,* and Δ*CrKP43-C*. Ultrastructure was observed under a transmission electron microscope, and cells in yellow boxes are enlarged for details.

Transmission electron microscopy (TEM) analysis was performed to investigate the effects of the *CrKP43* gene on cellular morphology and development of *C. chloroleuca*, and the results indicated that gene deficiency caused abnormal ultrastructure. Typical fungal cells were filled, showing the integrated cell wall and plasma membrane, organized cytoplasm, and mitochondria with well-defined envelopes, and other organelles were also intact. By contrast, the deletion of *CrKP43* had remarkable effects on cellular morphology (Fig. 4), including larger vacuoles and fewer organelles, indicating that the *CrKP43* gene was involved in the hyphal functions and activities of *C. chloroleuca*. Additionally, cell morphology of the complemented transformant Δ*CrKP43-C* showed similar results to those of the wild-type strain.

### CrKP43 is involved in antifungal activities of *C. chloroleuca*

**Fig 5:**
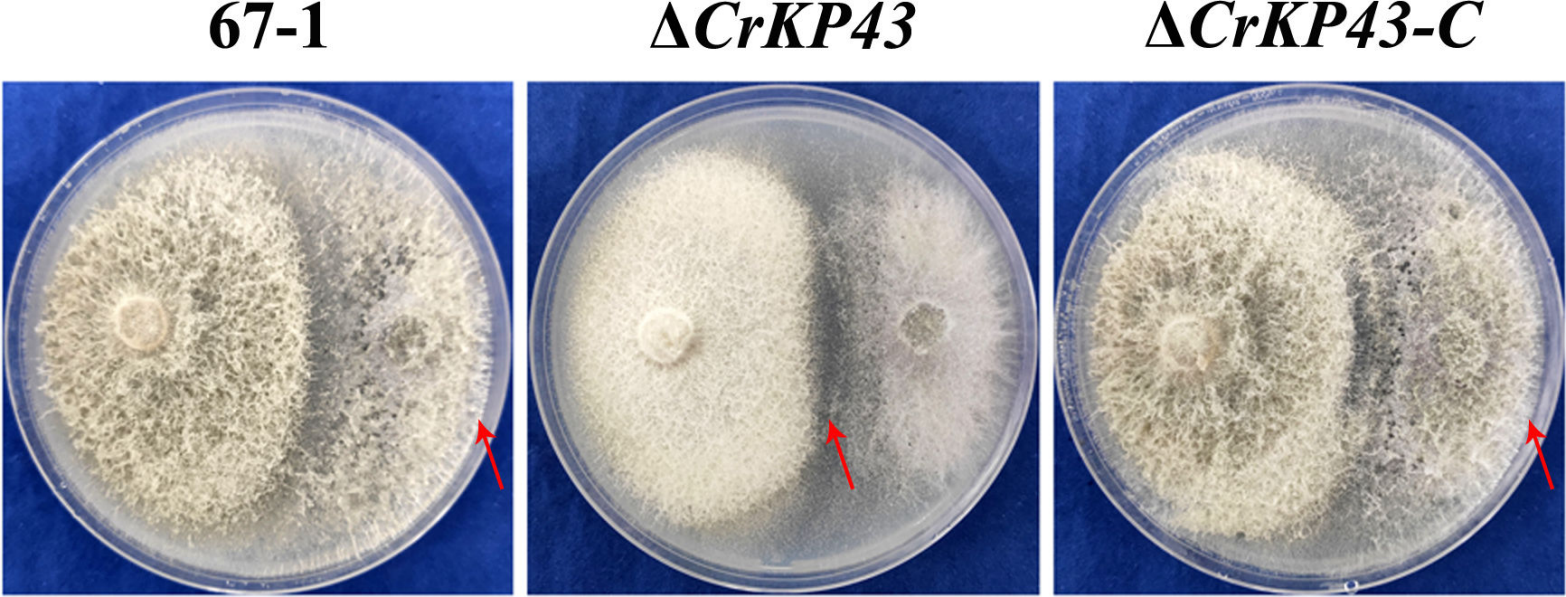
Impacts of *CrKP43* on the antagonistic activities of *C. chloroleuca* strains. Confrontation cultures of 67–1, Δ*CrKP43,* and Δ*CrKP43-C* against *F. oxysporum* f. sp. *cucumerinum* were assayed at 20 days post-inoculation. The red arrows indicate the hyphal extension distances of each strain toward the pathogenic fungus. All assays were performed in three replicates.

*C. chloroleuca* 67–1 displayed high antifungal activity against *F. oxysporum* f. sp. *cucumerinum in vitro*. However, when *CrKP43* was deleted, the effect was diminished. The inhibition rates of Δ*CrKP43* against the pathogenic fungus were 42.26%, markedly lower than that of the wild type (*P* < 0.05) after culturing for 20 days. The activity was recovered in the complemented strain Δ*CrKP43-C* to almost that of the wild type (Fig. 5).

### CrKP43 is essential for *C. chloroleuca* to mycoparasitize *S. sclerotiorum* sclerotia

**Fig 6:**
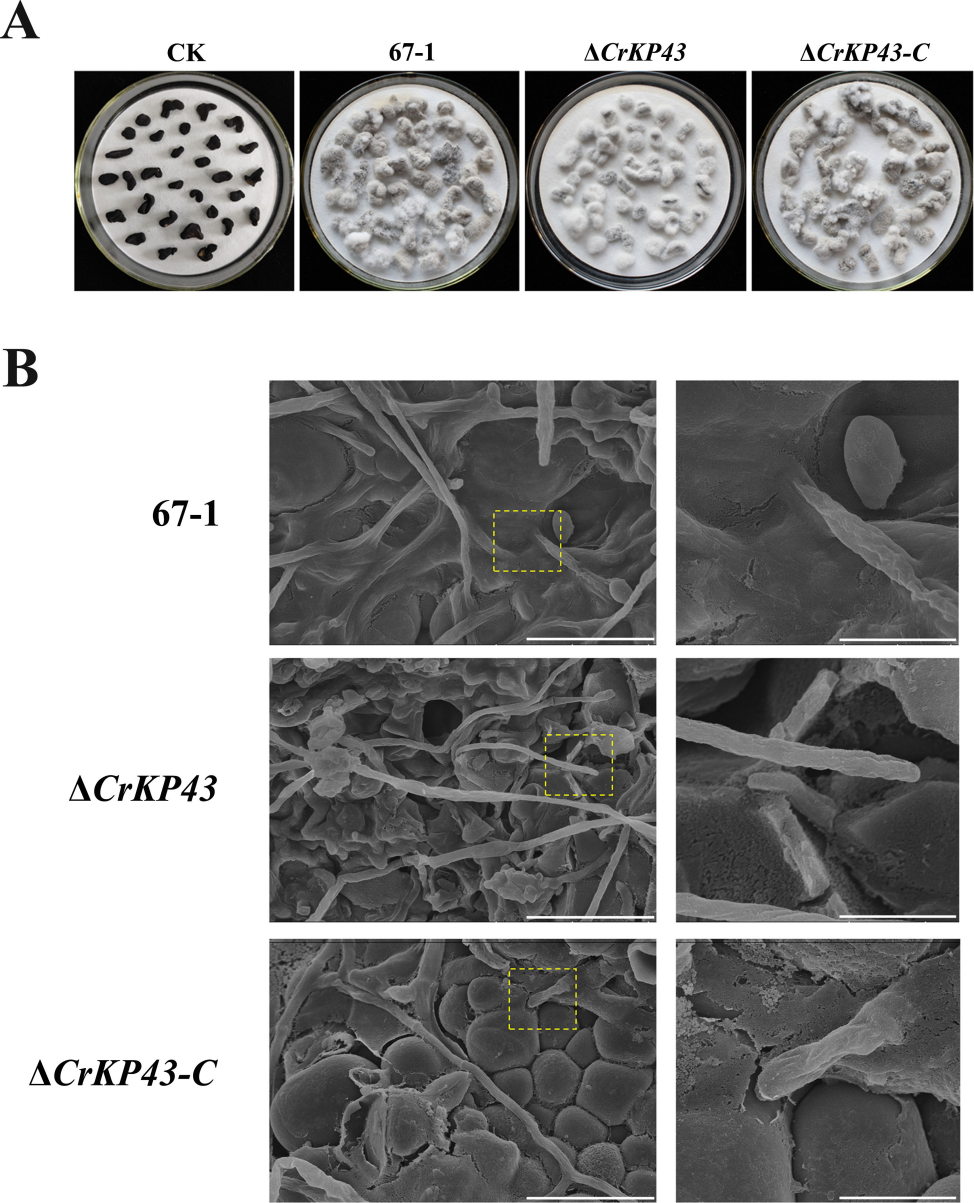
Mycoparasitism of *C. chloroleuca* strains to *S. sclerotiorum* sclerotia. (**A**) Sclerotia infected by different strains of *C. chloroleuca*. These images were captured after 7 days of incubation at 26°C. (**B**) Infection by *C. chloroleuca* 67–1, Δ*CrKP43,* and Δ*CrKP43-C* observed under a scanning electron microscope (bar = 20 µm). The parts in yellow boxes are enlarged for details (bar = 5 µm).

After 7 days of cultivation in moist conditions, the mycoparasitic ability of Δ*CrKP43* toward sclerotia was greatly reduced compared with that of the wild-type 67–1 and complemented strain Δ*CrKP43-C* (Fig. 6A). The sclerotia infected by the wild-type strain were completely softened and rotten, resulting in a high parasitic severity of grade 4. By comparison, those treated with the *CrKP43*-deficient mutant were fully covered with *C. chloroleuca* hyphae but remained relatively firm, equating to parasitic grade 3. Additionally, the mycoparasitic ability was recovered in the complemented strain, indicating that the *CrKP43* gene was involved in the mycoparasitism of *C. chloroleuca*.

Under a scanning electron microscope, the parasitic process and infection structures formed in *C. chloroleuca* isolates could be clearly observed. The hyphae of the wild-type strain grew and extended firmly on the surface of *S. sclerotiorum* sclerotia. More importantly, numerous infection points appeared, and degradation of *S. sclerotiorum* cell walls occurred. However, sparse mycelia with blunt ends were found on the sclerotia treated with Δ*CrKP43* mutants, and penetration to pathogen cells rarely occurred. The infection points are marked with yellow boxes and enlarged in Fig. 6B. When *CrKP43* was complemented, intensive invasion was achieved, similar to the wild type.

### CrKP43 is important for the biocontrol of *C. chloroleuca*

In pot experiments in the greenhouse, leaf lesions were observed in soybean seedlings after inoculation with *S. sclerotiorum* for 7 days. However, the seedlings treated with the biocontrol fungus 67–1 were much healthier and displayed less damage, consistent with excellent control efficacy against soybean *Sclerotinia* rot (Table 1). The control efficacies of *CrKP43*-deficient mutants decreased by 31.6% compared to 71.5% of the wild-type 67–1 (*P* < 0.05), while the efficiency regained in the complemented strains, demonstrating that *CrKP43* greatly enhanced the biocontrol efficacy of *C. chloroleuca*.

**TABLE 1:** Control efficacies of *C. chloroleuca* strains against soybean *Sclerotinia* stem rot^*a*^

Strain	Disease index	Control efficacy (%)
CK	65.2 ± 0.8 a	-
WT	18.6 ± 1.3 c	71.5 ± 0.7 a
Δ*CrKP43*	44.6 ± 1.0 b	31.6 ± 1.3 b
Δ*CrKP43-C*	21.8 ± 1.1 c	66.6 ± 0.9 a

^*a*^ Data are presented as means ± standard deviations of three replicates. Three mutants were tested for each treatment. The different letters in the columns indicate the significant differences according to Tukey’s tests (*P* < 0.05).

### *CrKP43* is differentially expressed when Δ*Crmapk* mycoparasitizes *S. sclerotiorum* sclerotia

**Fig 7:**
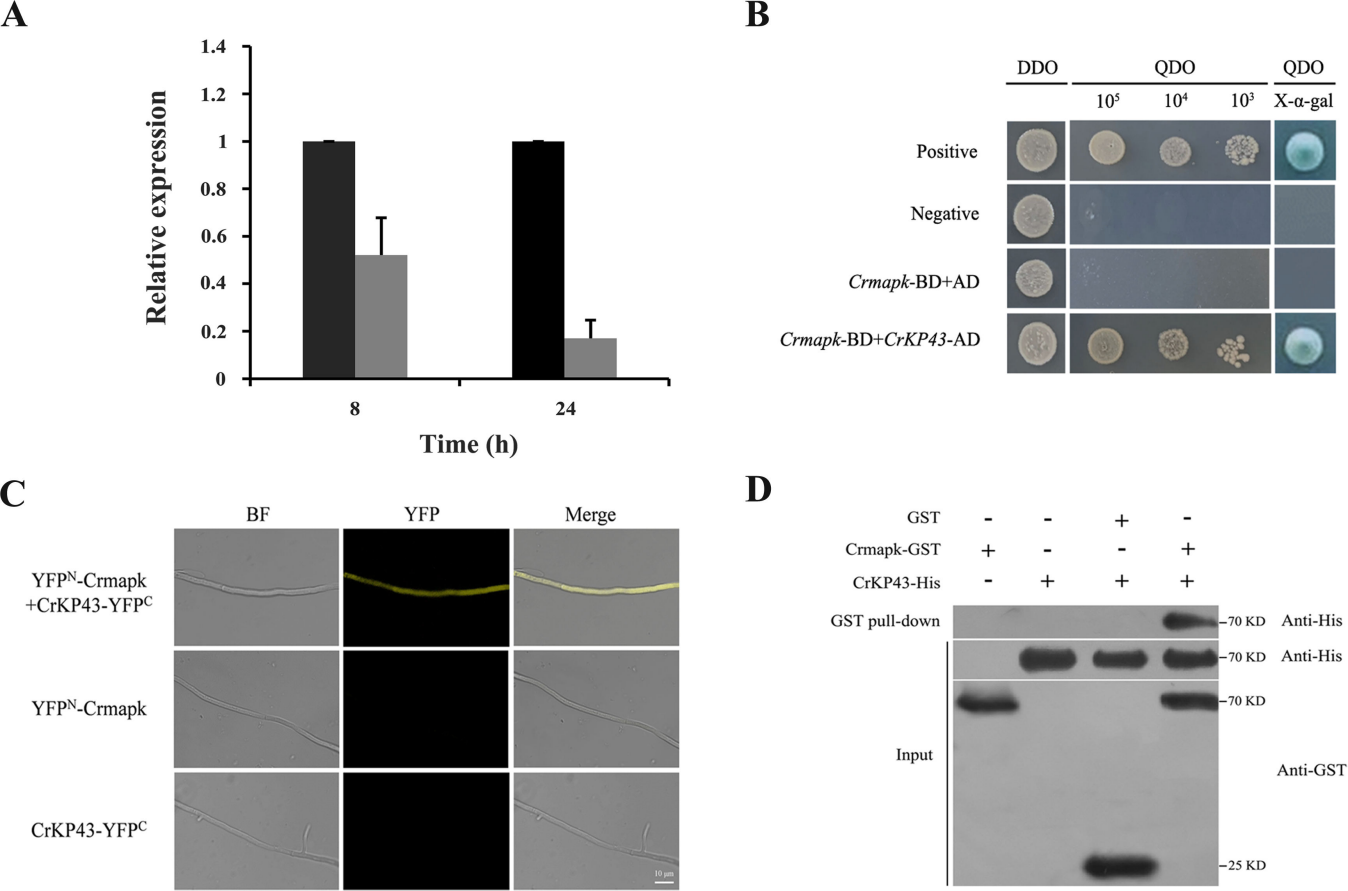
Verification of CrKP43 proteins interacting with Crmapk. (**A**) Expression levels of *CrKP43* in *Crmapk*-deficient mutants mycoparasitizing *S. sclerotiorum* sclerotia. The gray columns represent the fungal samples treated with fresh sclerotia, while the black columns represent the controls without sclerotia. Relative expression levels of *CrKP43* were calculated using the 2^–∆∆Ct^ method. The error bars show the standard deviations of three replicates. (**B**) Y2H assay. Yeast transformants expressing bait and prey vectors were incubated on the SD-Leu-Trp (DDO) plates and assayed on the SD-Ade-His-Leu-Trp (QDO) plates with X-α-gal. (**C**) BiFC assay *in vivo*. Transformants co-expressing YFP^N^-Crmapk and CrKP43-YFP^C^ were observed under a confocal fluorescence microscope. Bar = 10 µm. (**D**) GST pull-down assay *in vitro*. Proteins were pulled down using glutathione sepharose beads, and eluted samples were analyzed using Western blotting with anti-His and anti-GST antibodies.

In previous studies, we confirmed that the MAPK-encoding gene *Crmapk* was of great importance in the mycoparasitism of *C. chloroleuca*. By analyzing the transcriptome of Δ*Crmapk* mutants (29), the *CrKP43* gene was found differentially expressed accordingly (|Log2 FC| ≥ 1 and *P* ≤ 0.05). qRT-PCR verification showed that *CrKP43* expression was downregulated when Δ*Crmapk* parasitized sclerotia, and expression levels were 1.91- and 5.94-fold lower than those in the control at 8 h and 24 h, respectively (Fig. 7A), consistent with the above transcriptome data. The findings indicate that CrKP43 might be involved in mycoparasitism via the regulation of Crmapk.

### CrKP43 interacts with Crmapk in *C. chloroleuca*

Using the Y2H technique, the functional relationship of CrKP43 and Crmapk in *C. chloroleuca* was investigated, and the results showed that Crmapk might interact with CrKP43 (Fig. 7B). Furthermore, the interaction was confirmed by bimolecular fluorescence complementation (BiFC) assays *in vivo*, showing that the transformants co-expressing YFP^N^-Crmapk and CrKP43-YFP^C^ exhibited strong yellow fluorescent protein (YFP) signals in the cytoplasm (Fig. 7C), whereas no YFP signal was observed in the strains transformed with YFP^N^-Crmapk or CrKP43-YFP^C^. Furthermore, the interaction between Crmapk and CrKP43 was verified by the glutathione S-transferase (GST) pull-down method (Fig. 7D). Protein samples were pulled down using glutathione sepharose beads and further assessed by Western blotting with an anti-His antibody, yielding an appropriate-sized band for Crmapk-GST and CrKP43-His. Protein input samples were also assessed using anti-GST or anti-His as a reference.

## DISCUSSION

As a mycoparasite, *C. chloroleuca* can attack and kill plant pathogenic fungi (42 – 44). In contact with mycohosts or exposure to their signals, the expression of genes associated with the production of secondary metabolites, hydrolytic enzymes, and other secreted proteins is induced in *C. chloroleuca* (35, 45, 46). Proteases representing a very diverse group of hydrolases are involved in various cellular processes in organisms (1, 3, 47). In the current study, we found that the serine protease gene *CrKP43* was markedly upregulated and expressed both in the highly efficient *C. chloroleuca* isolate and in mutants lacking the MAPK gene *Crmapk* under the induction of *S. sclerotiorum* sclerotia. Additionally, CrKP43 was found to be associated with the growth, conidiation, and mycoparasitism of *C. chloroleuca*, possibly via MAPK pathways through interaction with Crmapk.

Mycoparasitism is one of the most important mechanisms for biocontrol fungi. Successful invasion of a mycoparasite is dependent on normal growth, conidiation, and mass production of infection structures that ensure the vitality and pathogenicity of the biocontrol fungus (34, 48, 49). In our study, *CrKP43*-deficient mutants exhibited greatly reduced conidiation of *C. chloroleuca* and caused abnormal hyphal morphology and ultrastructure, such as significantly enlarged vacuoles. Conidia attachment and germination are usually considered to indicate the start of mycoparasitism, but disruption of *CrKP43* significantly damaged fungal conidiation, consequently decreasing the penetration and mycoparasitic ability of *C. chloroleuca*. Fungal vacuoles are linked to a diverse range of cellular functions, including cellular homeostasis, degradation of intracellular components, and storage of ions and molecules (50). Therefore, we speculated that the *CrKP43* gene hindered hyphal functions and activities of *C. chloroleuca* by altering the morphology and levels of key components (e.g., amino acids and enzymes) of vacuoles. It would be interesting to further explore the roles of *CrKP43* in regulating fungal sporulation and vacuole production.

To infect and survive in hosts, mycoparasites need to degrade host cell walls, and hydrolytic enzymes are abundantly expressed during this process. In *C. chloroleuca* 67–1, 15 protease genes were found to be induced during colonization of *S. sclerotiorum* sclerotia (25). Troian et al. (22) found that the aminopeptidase-encoding gene *s9* was differentially expressed in *T. harzianum* during interaction with *S. sclerotiorum* and involved in parasitism of the antagonist. In extensive studies on *Trichoderma*, serine proteases, aspartyl proteases, and subtilisin-like proteases are involved in mycoparasitic actions (51 – 53). The diverse substrate specificities of proteases may act synergistically for efficient degradation of proteins anchored on host cell walls. Our current findings confirmed that deletion of *CrKP43* severely impaired the antagonistic activity and mycoparasitic ability of the biocontrol fungus and decreased its control efficacy against soybean *Sclerotinia* rot.

Mycoparasitism is a complex process involving multiple signal transduction pathways, including MAPK and cAMP pathways. When cells perceive external hosts or environmental stimuli, these signal pathways are initiated, and cellular responses are triggered (27, 36, 54). In eukaryotic organisms, MAPKs usually interact with other proteins to accomplish signal transmission. In a previous study, we identified the MAP kinase Crmapk orthologous to Fus3/Kss1 MAPK in *S. cerevisiae* and confirmed that it regulated the mycoparasitic ability and biocontrol efficiency of *C. chloroleuca* (29). Herein, the serine protease CrKP43 was confirmed to interact with Crmapk, and this interaction modulated mycoparasitic action in *C. chloroleuca*. To the best of our knowledge, this is the first report of an interaction between a serine protease and a MAP kinase in biocontrol fungi. The findings provide new insight into the mechanisms by which CrKP43 regulates mycoparasitism of *C. chloroleuca*.

According to the results of our study and the schematic diagram of the Fus3/Kss1-MAPK pathways in *S. cerevisiae* (27, 28, 55), we assume that the serine proteases will be induced once encounter with pathogenic fungi, sequentially affect biological processes such as growth and conidiation, and reduce the mycoparasitic ability and biocontrol efficiency of *C. chloroleuca* through regulation by Crmapk. This is supported to some extent by the transcriptome data showing that the *CrKP43* gene is differentially expressed both in the wild-type strain and Δ*Crmapk* mutant strains when parasitizing *S. sclerotiorum*.

In summary, we characterize the serine protease CrKP43 that regulates hyphal growth, conidiation, mycoparasitism, and biocontrol efficacy in *C. chloroleuca*, and demonstrate that CrKP43 may be involved in mycoparasitism of *C. chloroleuca* through regulation of Crmapk. Our findings reveal the functions and regulatory mechanisms of serine proteases in *C. chloroleuca* and lay a foundation to further explore the molecular mechanisms underlying mycoparasitism of *C. chloroleuca*. The study will also facilitate the development of highly efficient fungal biocontrol agents.

## MATERIALS AND METHODS

### Fungal strains

*C. chloroleuca* 67–1 (ACCC 39160) was originally isolated from a vegetable yard in Hainan Province, China, using the sclerotia-baiting method (56). *S. sclerotiorum* Ss-H (ACCC 39161) was separated from sclerotia-infected soybean stems in a field in Heilongjiang Province, China. *F. oxysporum* f. sp. *cucumerinum* foc-3b was isolated from an infected cucumber root in a field in Hebei Province, China (57). All strains were cultured routinely on PDA plates at 26°C and maintained at 4°C.

### Bioinformatics analysis of CrKP43

The DNA sequence of *CrKP43* was obtained from the draft genome sequence of *C. chloroleuca* 67–1 (58). NCBI (http://www.ncbi.nlm.nih.gov/) and UniProt (http://www. uniprot.org/blast/) were used for BLASTp analysis. Functional domains of *CrKP43* were predicted using SMART (http://smart.embl.de/). The Clustal X program (59) was used for amino acid alignments. A phylogenetic tree was constructed by MEGA 7.0 (60) using the maximum likelihood method with 1,000 bootstrap replicates.

### qRT-PCR analysis of *CrKP43*

Total RNAs of *C. chloroleuca* 67–1 mycoparasitizing *S. sclerotiorum* sclerotia were extracted at different stages (8 h, 24 h, and 48 h) using TRIzol reagent (Invitrogen, CA, USA) according to standard procedures. RNase-free DNase I (Invitrogen) was used to eliminate DNA contamination. Reverse transcription was performed using a cDNA FastQuant RT Kit (Tiangen, Beijing, China).

Gene expression of *CrKP43* in *C. chloroleuca* was monitored by qRT-PCR using a Bio-Rad IQ 5 Real-Time System (Bio-Rad, CA, USA) and SYBR Premix Ex Taq (TaKaRa, Dalian, China). The primers (CrKP43-F/CrKP43-R) were designed using Primer3 (https://www.yeastgenome.org/primer3) (Table S1), and the elongation factor gene *EF1* (GenBank accession number: KP274074) served as an internal reference gene to normalize gene expression in the parasitic process (25, 61). The mycelial samples of *C. chloroleuca* 67–1 without induction of sclerotia acted as the control. Relative expression levels of *CrKP43* were calculated using the 2^–∆∆Ct^ method, and three biological replicates were conducted for each sample.

Gene expression of *CrKP43* in the *Crmapk*-deficient mutant when mycoparasitizing *S. sclerotiorum* sclerotia was also monitored as described above.

### Gene deletion and complementation

The plasmid pKH-KO containing two uracil-specific excision reagent (USER) cloning sites (USC1 and USC2) on either side of the hygromycin resistance gene *hph* was used to construct a *CrKP43* disruption vector (62). Upstream and downstream flanking sequences of *CrKP43* were amplified using the primer pairs CrKP43-UF/CrKP43-UR and CrKP43-DF/CrKP43-DR, respectively, and cloned into two USC sites using the USER-friendly cloning method to generate *CrKP43*-deletion vector pKH-KO-CrKP43.

To construct a complementation vector, the full-length complement fragment of *CrKP43* containing a native promoter, as well as protein-coding and terminator regions, was amplified from the 67–1 genomic DNA and cloned into the pKN vector carrying the G418 resistance gene *neo* (48). The resulting gene deletion and complementation vectors were transformed into the protoplasts of 67–1 and Δ*CrKP43* strains, respectively, and the gene-deficient and complementary mutants were generated using the strategy described previously (63). The primers (Table S1) were designed, and the mutants were confirmed by PCR and DNA sequencing. Furthermore, the expression levels of *CrKP43* in the wild type, deletion, and complementation strains were tested using RT-PCR with the primers CrKP43-F/CrKP43-R and reference gene *EF-1* (Table S1) (25, 61).

### Growth and conidiation of *CrKP43* mutants

The phenotypes of *C. chloroleuca* 67–1, Δ*CrKP43,* and Δ*CrKP43-C* strains were characterized on plates. Agar blocks (3-mm diameter) of the strains cultured on PDA for 2 days were cut from the edges of the colonies and inoculated on the centers of the PDA plates. The isolates were cultured at 26°C in the dark in an incubator, and extensions of the colonies were measured daily. After 15 days, the yielded spores were washed with 5-mL sterile distilled water and counted under a BX41 microscope (Olympus, Tokyo, Japan) using a hemocytometer.

### Stress tolerance of *CrKP43* mutants

The stress responses of *C. chloroleuca* isolates to osmolarity, oxidative stress, cell membrane stress, and cell wall stress were determined on the PDA media amended with different stress agents (1-M NaCl, 1-M KCl, 1-M glycerin, 1-M sorbitol, 20-mM H_2_O_2_, 0.03% SDS, 0.3-mg/mL Congo Red). The wild-type, Δ*CrKP43,* and Δ*CrKP43-C* strains of *C. chloroleuca* 67–1 were inoculated on the centers of agar plates and incubated at 26°C. After 10 days, fungal growth was measured. All assays were repeated three times.

### Cellular morphology of *CrKP43* mutants

The ultrastructure of *C. chloroleuca* isolates was observed using TEM. The conidia of the wild-type, Δ*CrKP43,* and Δ*CrKP43-C* strains were collected from the 14-day-old PDA plates, inoculated into the PDB medium, and cultured at 26°C with shaking at 180 rpm for 24 h. Fresh mycelia were collected, washed, and fixed with 2.5% glutaraldehyde in 0.1-M phosphate-buffered saline (PBS; pH 7.4) at 4°C overnight. The samples were washed three times with the PBS buffer prior to fixing in 1% osmium tetroxide buffered with 0.1-M cacodylate (pH 7.0) at 4°C overnight and then dehydrated in a graded series of ethanol and infiltrated with a series of epoxy resin in epoxy propane prior to embedding in Epon-812 resin. Ultrathin sections of each sample were cut with an EM UC6 ultramicrotome (LEICA, Wetzlar, Germany) and visualized under an H-7500 transmission electron microscope (Hitachi, Tokyo, Japan) operating at 80 kV.

### Antifungal activities

The antagonistic activities of the wild-type strain and mutants of *C. chloroleuca* against *F. oxysporum* f. sp. *cucumerinum* (*Foc*) were tested on 9-cm PDA plates. A 3-mm agar plug of each strain was placed 2 cm from the edge of the plate and cultured at 26°C for 5 days. A plug of *Foc* was then placed equidistantly from the other side. After confrontation culture at 26°C for 20 days, the distance of hyphal extension onto the colony of the pathogenic fungus was measured (64 – 66). All assays were performed in three replicates.

### Mycoparasitic ability against *S. sclerotiorum* sclerotia

The sclerotia with uniform size were surface-sterilized with 1% NaClO for 3 min, washed three times with sterile water, and then immersed in spore suspensions of the wild-type 67–1, Δ*CrKP43,* and Δ*CrKP43-C* strains at a concentration of 1 × 10^7^ spores/mL for 10 min. Sclerotia were picked and placed onto a piece of wet sterile filter paper in a Petri dish (diameter 9 cm) and incubated at 26°C. Treatment with sterile water was used as a control. After 7 days, the parasitic severity of sclerotia was examined using a BX41 inverted microscope (Olympus) based on a four-grade scale (0 = no *C*. *chloroleuca* hyphae detected on the surface of sclerotia; 1 = loose *C*. *chloroleuca* hyphae extended to the sclerotia; 2 = sclerotia covered with *C. chloroleuca* hyphae but not softened; and 3 = sclerotia covered with *C. chloroleuca* hyphae and exhibiting soft rot) (49). A total of 30 sclerotia were tested for each treatment, with three replicates. Additionally, the sclerotia parasitized by the transformants for 48 h were cut into ~0.5-mm slices, fixed in 2.5% glutaraldehyde at 25℃ in the dark for 48 h, and stored at 4℃ before observation. The specimens were gently dried using an EM CPD030 instrument (LEICA) and coated with gold powder. The mycoparasitic abilities of different strains of *C. chloroleuca* against *S. sclerotiorum* sclerotia were detected under an S-570 scanning electron microscope (Hitachi) with an accelerating voltage of 10 kV. Three replicates were performed for each sample.

### Control efficacy against soybean *Sclerotinia* rot in greenhouse experiments

The control effects of *CrKP43* on soybean *Sclerotinia* rot were tested in pots in a greenhouse. Soybean seeds (Zhonghuang 13; Institute of Crop Sciences, CAAS, China) were sown in sterile soil in plastic pots (11-cm diameter). When nine compound leaves had grown, seedlings were sprayed with 100-mL spore suspension (1 × 10^7^ spores/mL) from each strain. After drying for 2 h, an equivalent amount of *S. sclerotiorum* mycelial suspension was inoculated onto the leaves. The plants treated with sterile water followed by the pathogen served as the control, and 12 pots were tested for each isolate. The greenhouse was maintained at 26–28°C and 60% relative humidity, and all pots were arranged randomly. After 7 days, the disease severity of *Sclerotinia* rot was scored using grades 0 to 4 according to the percentage of lesions on the soybean leaves (0 = no symptoms on the soybean leaves; 1 = less than 10% lesions on the soybean leaves, 2 = 10%–30% lesions on the soybean leaves; and 3 = 30%–50% lesions on the soybean leaves; 4 = over 50% lesions on the soybean leaves). All unfolded compound leaves were checked, and three replicates were performed for each treatment.

### Y2H assays

From the Y2H library of *C. chloroleuca* constructed previously, S8 serine protease CrKP43 was found to be associated with the MAP kinase Crmapk. To confirm the interaction between the two proteins, the coding sequence (CDS) of *Crmapk* and *CrKP43* genes was amplified using 67–1 cDNA as a template. The *CrKP43* fragment was cloned into pGADT7 as the prey vector, and *Crmapk* was cloned into pGBKT7 (Clontech, Mountain View, CA, USA) as the bait vector using the corresponding primers (Table S1). After sequencing, the pairs of Y2H plasmids were co-transformed into the *S. cerevisiae* strain Y2H Gold using PEG/LiAc-mediated transformation. The plasmids pGBKT7-53 and pGADT7 served as positive controls, while the plasmids pGBKT7-Lam and pGADT7 served as negative controls. The transformants were cultured on a synthetic medium (SD) lacking Leu and Trp (DDO) at 30°C for 3–5 days and then transferred to SD plates lacking His, Leu, Trp, and Ade (QDO) to validate the interaction of CrKP43 and Crmapk. Three independent experiments were performed.

### BiFC assays

The YFP^N^-Crmapk plasmid was constructed by cloning the *Crmapk* gene with its native promoter into the pHZ65 vector harboring YFP^N^ and the hygromycin B resistance cassette. Similarly, the CrKP43-YFP^C^ plasmid was constructed by cloning the *CrKP43* gene into the pHZ68 vector carrying YFP^C^ and the zeocin resistance cassette. The pairs of constructs (YFP^N^-Crmapk and CrKP43-YFP^C^) were transformed into the protoplasts of *C. chloroleuca* 67–1, and the transformants were selected using both hygromycin and zeocin, as confirmed by PCR. The recombination plasmids YFP^N^-Crmapk or CrKP43-YFP^C^ were individually transformed into 67–1, and the resultant transformants served as negative controls. The YFP signals in mycelia grown in PDB for 48 h were examined under an LSM980 confocal fluorescence laser scanning microscope (Zeiss, Gottingen, Germany).

### GST pull-down assays

The DNA fragment of Crmapk was cloned into the vector pGEX-4T-1 (GE Healthcare, Chicago, IL, USA) to generate the Crmapk-GST fusion protein. CrKP43 was incorporated into the pCZN1 vector (Zoonbio, Nanjing, China) to express the CrKP43-His fusion protein. GST, Crmapk-GST, and CrKP43-His plasmids were expressed in *Escherichia coli* BL21 cells (Sangon, Shanghai, China). The cells were lysed in lysis buffer [50-mM Tris pH 8.0, 50-mM NaCl, 1-mM phenylmethanesulfonyl fluoride (PMSF)] with a sonicator (Scientz, Ningbo, China) and centrifuged at 13,000 g for 10 min. The supernatants were transferred to a 1.5-mL tube and stored at −70°C. The GST and Crmapk-GST supernatants were mixed with 30-µL glutathione sepharose beads (GE Healthcare) and incubated at 4°C for 2 h, and the recombinant Crmapk-GST and GST bound to beads were incubated with the *E. coli* cell lysate containing CrKP43-His at 4°C. After 4 h, the beads were washed five times with buffer (50-mM Tris pH 8.0, 50-mM NaCl, 1-mM PMSF, 1% Triton X-100), and the eluted proteins were analyzed by immunoblotting with monoclonal anti-His and monoclonal anti-GST antibodies (Abcam, Cambridge, UK).

### Statistical analysis

Statistical software SPSS 2.0 (SPSS Inc., Chicago, IL, USA) was used for the analysis of variance (ANOVA). Statistical tests were carried out for multiple comparisons using Tukey’s test, and *P* < 0.05 was considered statistically significant.

## SUPPLEMENTAL MATERIAL

Supplemental material spectrum.02448-23-s0001.docx Fig. S1 and S2; Table S1.

ASM does not own the copyrights to Supplemental Material that may be linked to, or accessed through, an article. The authors have granted ASM a non-exclusive, world-wide license to publish the Supplemental Material files. Please contact the corresponding author directly for reuse.
