# Supplementary material for: Serine protease CrKP43 interacts with MAPK and regulates fungal development and mycoparasitism in Clonostachys chloroleuca
Source: Microbiol Spectr. 2023 Oct 13;11(6):e02448-23. doi: 10.1128/spectrum.02448-23 (PMC10715147; doi:10.1128/spectrum.02448-23)
Supplement: Supplemental material — Fig. S1 and S2; Table S1. [file spectrum.02448-23-s0001.docx]

**SUPPLEMENTARY DATA:**


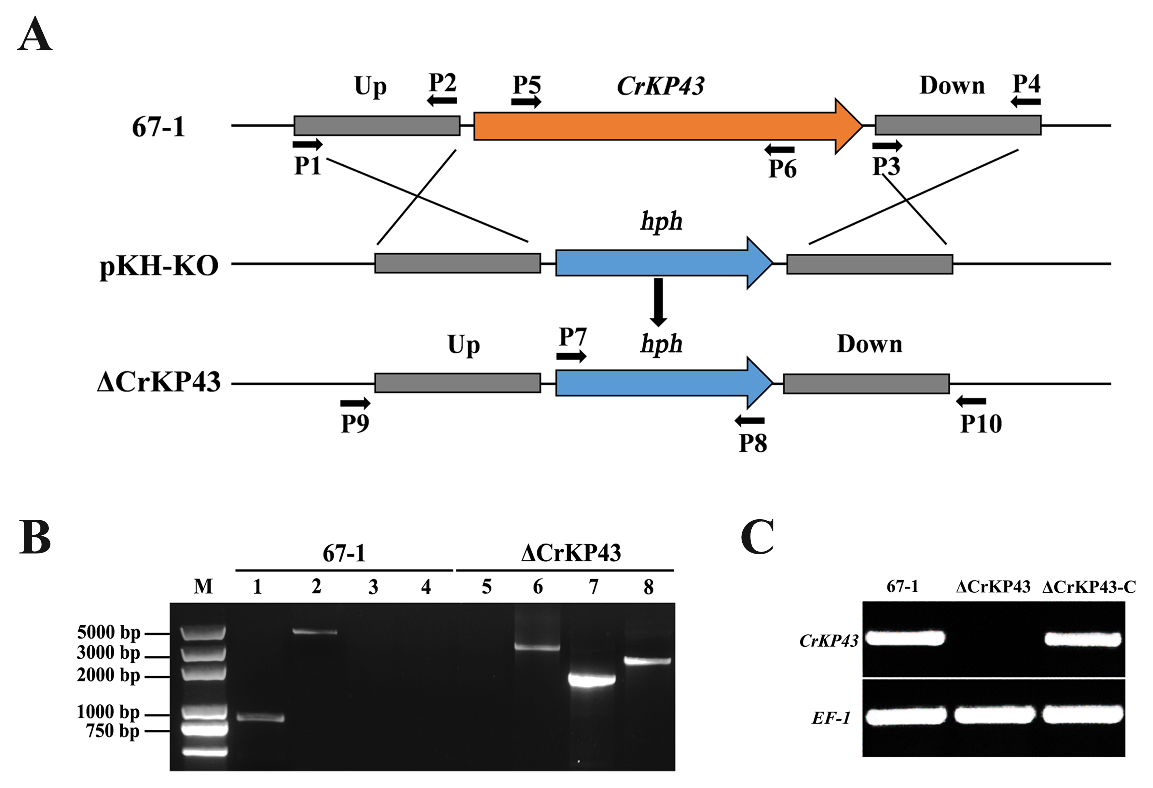


**FIG** **S1** Construction of deletion vector of *C. chloroleuca* and confirmation of *CrKP43*-dedicient mutants. (A) Schematic diagram of the gene disruption strategy. The hygromycin resistance cassette (*hph*) was cloned into the corresponding site of vector pKH-KO-CrKP43, replacing a 2060 bp *CrKP43* open reading frame. The annealing sites of the primers are indicated by short black arrows. (B) PCR verification of *C. chloroleuca* 67-1 and gene deletion mutants. Lanes 1−4, strain 67-1; Lanes 5−8, Δ*CrKP43* mutants. The primers P5/P6 (CrKP43-in-F/R), P9/P10 (CrKP43-yz-F/R), P7/P8 (HPH-F/R) and P9/P8 (CrKP43-yz-F/HPH-R) were used, respectively. (C) RT-PCR analysis of *CrKP43* gene expression in 67-1, Δ*CrKP43* and Δ*CrKP43-C* strains using specific CrKP43-F/R primers (**Table** **S1**).


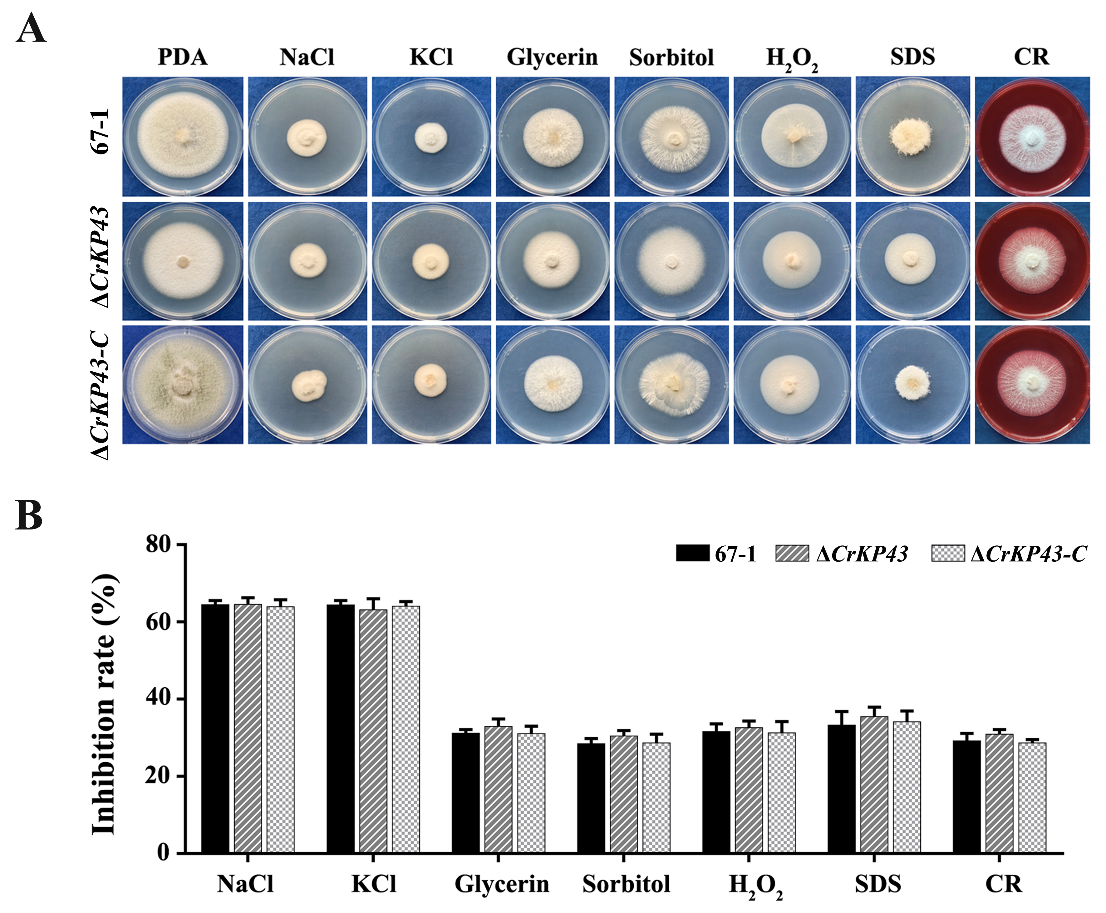


**FIG** **S2** Sensitivities of *C. chloroleuca* 67-1, Δ*CrKP43* and Δ*CrKP43-C* to environmental stresses. (A) Growth of *C. chloroleuca* strains on PDA plates containing 1 M NaCl, 1 M KCl, 1 M glycerin, 1 M sorbitol, 20 mM H_2_O_2_, 0.03% SDS and 0.3 mg/ml Congo Red (CR) after 10 days of incubation at 26°C. (B) Inhibition of mycelial growth compared with those on PDA plates without stress agents. Results are means of three mutants, and means and standard errors were calculated from three independent replicates. Statistical analyses were carried out using Tukey tests for multiple comparisons, and asterisks indicate signiﬁcant differences (*P* < 0.05).

**Table S1 Primers used in this study**

| **Primer name** | **Sequence (5’**−**3’)** | **Purpose** |
| --- | --- | --- |
| CrKP43-F | CACCCAATGCATCCCTTGTC | qRT-PCR primer of *CrKP43* |
| CrKP43-R | CTCACCATCGTTTCCAGCAG |  |
| EF1-F | TCGATGTCGCTCCTGACT | qRT-PCR Primer of *EF1* |
| EF1-R | AGCGTGACCGTTTATTTGA |  |
| CrKP43-UF | GGTCTTAAU TTCTAGGCTTCACTCCGCTG | Amplification of *CrKP43* upstream sequence |
| CrKP43-UR | GGCATTAAU ATGAGAACAAGAGAGGGCAAA |  |
| CrKP43-DF | GGACTTAAU GCATGGCTCGATGAATTTCTG | Amplification of *CrKP43* downstream sequence |
| CrKP43-DR | GGGTTTAAU GCCTCTGTCGTCAACCATGA |  |
| CrKP43-IN-F | GCGTCTTTGTGTCCGAGTAC | Validation of *CrKP43* deletion |
| CrKP43-IN-R | AACAAGACCCCACGAATTGC |  |
| HPH-F | TGGAGCTAGTGGAGGTCAACA | Amplification of *hph* sequence |
| HPH-R | CGGTCGGCATCTACTCTATTC |  |
| CrKP43-YZ-F | CAGAGCTGGATGTCGGTGA | Validation of *CrKP43* deletion |
| CrKP43-YZ-R | GCCCACCAATCTTATGCAGC |  |
| CrKP43-COMF | CCCCCGGGCTGCAGGAATTCATGCAGTCATCTTCCACCGG | *CrKP43* complementation |
| CrKP43-COMR | TCGACGGTATCGATAAGCTTGATGTTTGGGTGGATAGCGG |  |
| Crmapk-F | CATGGAGGCCGAATTCATGTCTCGATCAACTCAGCCCAGC | Construction of pGBKT7-Crmapk |
| Crmapk-R | GCAGGTCGACGGATCCTCATCGCATGACCTCCTGGTAGAT |  |
| CrKP43-AD-F | GGAGGCCAGTGAATTCATGTCTTCCCGACGTCCACTCAGG | Construction of pGADT7-CrKP43 |
| CrKP43-AD-R | CGAGCTCGATGGATCCTCACTCATAAAGTCCCCAGGTTAC |  |
| Crmapk-65-F | CAGATCTTGGCTTTCGTAGGAACCCAATCTTCAATGTCTCGATCAACTCAGCC | Construction of YFP^N^-Crmapk |
| Crmapk-65-R | GCTCACCATCGTGGCGATGGAGCGTCGCATGACCTCCTGGTAGA |  |
| CrKP43-68-F | CAGATCTTGGCTTTCGTAGGAACCCAATCTTCAATGTCGCAGGCAGCCTTTGG | Construction of CrKP43-YFP^C^ |
| CrKP43-68-R | GTTCGGGATCTTGCAGGCCGGGCGCTCATAAAGTCCCCAGGTTAC |  |
